# Supplementary material for: Interregional circulation of credit resources and company innovation
Source: PLoS One. 2024 Aug 20;19(8):e0309022. doi: 10.1371/journal.pone.0309022 (PMC11335111; doi:10.1371/journal.pone.0309022)
Supplement: Attachment — Submitted filename: Response to Reviewers.docx [file pone.0309022.s003.docx]

Dear Reviewer(s),

Thanks for your careful comments. We have attached great importance to them. Recently, the paper has been revised and submitted. The responses to your comments and the specific changes are presented as follows.

**Responses to Reviewer 1**

**Comment:**

The author has taken my comments into account. The manuscript meets the journal's requirements. I recommend the article for publication.

**Response:**

Thanks for your positive feedback on the manuscript. We appreciate you taking the time to provide constructive comments during the review process. It has helped us to improve the quality and clarity of the work.

**Responses to Reviewer 2**

**Comment:**

Required changes are met. The checking of English should be verfied. The writing style must be also checked by the journal.

**Response:**

We have adopted proofreading and editing services. Two editors from Cambridge Proofreading LLC have corrected the grammar and improved the readability of the text. The proofreading and editing certificates have been submitted to the system as separate files labeled 'Other'.

Moreover, we have made some adjustments to ensure the manuscript meets PLOS ONE's style requirements. We have checked the heading type and font. We have used the PACE tool to check the figure and convert it to the accepted format. Then, we have uploaded the figure separately. Consistent with the requirements, the manuscript can be presented in double-spaced paragraph format. Additionally, we have changed the in-text citations from the hyphen (-) to the en-dash (–), e.g., [1-2] -> [1–2]. As for references, the journal name should be used as an abbreviation. We have modified it. The Supporting information is required to be listed at the end of the manuscript in the template file. But it appears before the References in the published articles. After careful consideration, we ensure that the revised manuscript meets the requirements of the PLOS ONE style templates. The templates can be found at the links below.

https://journals.plos.org/plosone/s/file?id=wjVg/PLOSOne_formatting_sample_main_body.pdf

https://journals.plos.org/plosone/s/file?id=ba62/PLOSOne_formatting_sample_title_authors_affiliations.pdf.

We refer to published literature for some formats not mentioned in the template files. For example, The first letter is capitalized for “Supporting information”, while all words have their first letters capitalized for “Author Contributions”.

Thanks for your valuable feedback. It enhances the clarity and comprehensiveness of our research. It also assists us in fulfilling the necessary requirements of the journal more effectively. We sincerely hope that the revised manuscript can be accepted for publication.

Kind regards,

Xinglin Liu
